# Adaptation of NS cells growth and differentiation to high-throughput screening-compatible plates

**DOI:** 10.1186/1471-2202-11-7

**Published:** 2010-01-19

**Authors:** Alessia Garavaglia, Alessia Moiana, Stefano Camnasio, Daniele Bolognini, Roberto Papait, Dorotea Rigamonti, Elena Cattaneo

**Affiliations:** 1 Dialectica S.r.l., c/o Nerviano Medical Sciences, V.le Pasteur 10, 20014 Nerviano MI, Italy; 2 Centre for Stem Cell Research & Department of Pharmacological Sciences University of Milano, Via Balzaretti 9, 20133 Milano, Italy

## Abstract

**Background:**

There is an urgent need of neuronal cell models to be applied to high-throughput screening settings while recapitulating physiological and/or pathological events occurring in the Central Nervous System (CNS). Stem cells offer a great opportunity in this direction since their self renewal capacity allows for large scale expansion. Protocols for directed differentiation also promise to generate populations of biochemically homogenous neuronal progenies. NS (Neural Stem) cells are a novel population of stem cells that undergo symmetric cell division in monolayer and chemically defined media, while remaining highly neurogenic.

**Results:**

We report the full adaptation of the NS cell systems for their growth and neuronal differentiation to 96- and 384-well microplates. This optimized system has also been exploited in homogeneous and high-content assays.

**Conclusions:**

Our results show that these mouse NS cells may be suitable for a series of applications in high-throughput format.

## Background

Highly-informative automated assays that monitor cell morphology, proliferation, death, motility and differentiation offer, nowadays, a great opportunity for the discovery of new gene functions, the study of molecular networks, the identification and validation of disease-relevant drug targets as well as the selection of pharmacologically active compounds. These assays are crucial for the obtainment of rapid, reliable and reproducible results. However, biological systems inevitably bring along some disadvantages with respect to a pure biochemical assay. Therefore one aim is to optimize the number and quality of the information gained from the selected cell model. Generally, the closest the cells employed recapitulate their *in vivo *counterparts, the more reliable the obtained data are for a given situation or pathology.

Cells for drug discovery research are typically obtained from primary tissues, genetically transformed or immortalized tumor cells [1]. More recent stem cell technologies may offer a number of important advantages, including the availability of an indefinite and reproducible source of relevant cells, the possibility to grow them close to biological and biochemical homogeneity and the capability to direct their differentiation toward the various mature cell types present in the body organism, including neurons. These benefits are to be taken into particular consideration especially when dealing with central nervous system (CNS) cells and related disorders, where the need for relevant cellular models is justified also by the difficulty in obtaining the relevant cells from fresh tissue. Moreover, improved *in vitro *models based on physiologically relevant neural cells may also presumably result in more cost-effective assays.

Furthermore, the employment of different neural stem cell systems and of their functionally mature neuronal progeny in screening assays may also open the possibility of a broader cell system biology approach, able to cast a much wider net than target-by-target approaches, increasing the possibility to identify pharmacologically active agents. In fact, many potential drugs do not bind to a single specific target but exert their effect through pathway mediated actions and these can be identified only by complex biological screens [2,3].

One available neural stem cell system is represented by the neurospheres culture which are identified as floating multicellular clusters that proliferate in the presence of epidermal growth factor (EGF) and/or fibroblast growth factor 2 (FGF2) [4,5]. Neurospheres have been demonstrated valuable for a number of approaches [6,7]. They have also been used in a screening application, searching for growth inhibitors [8] however some of the characteristics of the neurospheres culture make them not the elective method for screening applications. In fact, their composition is highly heterogeneous being the growing cell population characterized by progenitor cells, astroblasts, neuroblasts and differentiated progeny [9].

A possible source of interesting stem cells for drug screening studies is represented by the Embryonic Stem Cells (ESC). These cells allow the obtainment of transient populations of progenitors that divide *in vitro *and that can be then differentiated into a large number of different cell types [10]. Desbordes et al [11] have succeeded in the attempt to apply human ESCs (hESCs) to high-throughput screening (HTS) assays conducted in 384-well microplates. By employing the pluripotency factor Oct4 as a primary read-out for hESCs differentiation combined with a fully automated high-throughput laser-scanning confocal microscopy screening, a number of chemical compounds have been identified for their ability to influence hESCs maturation. However, as the authors themselves highlight, hESCs will become suitable for routine HTS applications only when the variability in the quality of hESCs maintained in bulk cultures will be overcome [12].

Our work focused on the characterization and implementation of a mouse derived NS cells system which can be long-term propagated in the absence of unwanted differentiation. NS cells grow homogeneously in monolayer and show a high potential to give rise to functionally mature neurons even after long term expansion, which may be exploited for screening applications. These cells can be generated from mouse embryonic stem cells (mES) or from fetal and adult nervous tissues [13,15], as well as derived from human fetal brain tissues [13,16]. NS-like cells capable of growing in monolayer have been recently obtained also from hESCs [17]. NS cells undergo sustained symmetrical self renewal in response to FGF2 and EGF, independently from any specific cellular niche [13]. Moreover, they display characteristics of neurogenic radial glia, since they are immunopositive for Nestin, RC2, Pax6, Vimentin, BLBP and GLAST and show interkinetic nuclear migration, a feature of neuroepithelial and radial glia cells *in vivo *[13]. Importantly, these stem cells are able to generate neurons, glial cells and oligodendrocytes and retain neurogenic potential after extensive *in vitro *expansion [13,18]. More recently, our group has developed a neuronal differentiation protocol which gives rise to 80-85% neurons from ES-derived NS cells, out of which approximately 70% are functionally active [19]. When applied to NS cells derived from the adult Sub Ventricular Zone (SVZ) of the mouse, a modification of this protocol generates a cellular population of which 65% are MAP2+ cells co-expressing GABA, GAD65/67, calbindin and parvalbumin and generating action potentials after 21 days *in vitro *differentiation [15].

Here we demonstrate that it is possible to grow these mouse NS cells in microplates and to monitor their proliferation rate and propensity to cell death using homogenous assays. Moreover, we confirm the capacity of adult SVZ-derived NS cells to undergo neuronal differentiation in microplate culture conditions (96- and 384-well microplates) without any reduction in the efficiency with respect to what observed in 24-well dishes [15]. We also report the application of homogenous assays to evaluate caspases 3/7 activity and cAMP levels in self-renewing NS cells and mature neurons, both using fixed and alive cells, with the incomparable advantage of opening to the possibility of monitoring specific patho-physiological parameters in well characterized stem cell-derived neuronal populations.

## Methods

### Chemicals

Forskolin (Tocris#1099), H_2_O_2 _(Sigma Aldrich#H1009), Ro 20-1724 (Sigma Aldrich #B8279), Staurosporine (Sigma Aldrich#S4400).

IBMX (Sigma Aldrich#I7018), NECA (Tocris#1691), CGS21680 (Tocris#1063), SCH58261 (Tocris#2270), SKF 38393 (Sigma Aldrich#D-047), quinpirole (Sigma Aldrich#Q-102), serotonine (Sigma Aldrich#H-9523), buspirone (Tocris#0962), RS67506 (Tocris#0990), acetylcholine (Sigma Aldrich#A2661), GABA (Sigma Aldrich#A5835).

### Cell culture

The obtainment of LC1 cells, a mouse ESC-derived NS cell line, is described in Conti et al, 2005 [13]. The adult SVZ-derived NS cells line aNS-1 was derived from the SVZ of a 2-months-old wild-type (CD1 strain) mouse, as previously reported [14].

NS cells were routinely cultured on Iwaki plasticware (Bibby, Italy) in NS proliferation medium, composed by Euromed-N medium (Euroclone) supplemented with N2 supplement (Gibco, Invitrogen, Italy), 10 ng/ml EGF (Peprotech Inc.) and 10 ng/ml FGF-2 (Peprotech Inc.). Detailed protocols for routine handling of aNS-1 cells are available elsewhere [14,15].

### Differentiation of NS cells

LC1 cells were differentiated using an optimized protocol in serum-free conditions [19]. Briefly, proliferating cells were collected following Accutase treatment and then plated on untreated plastic dishes in Euromed-N medium (EuroClone) medium supplemented with 5 mg/mL penicillin/streptomycin (Gibco, Invitrogen), 5 mg/mL L-Glutamine (Gibco, Invitrogen), 1% B27 (Gibco, Invitrogen), 0.5% N2 (Gibco, Invitrogen) and 10 ng/ml FGF2 (Peprotech, Tebu-Bio). After 3 days at 37°C cultures were dissociated with Accutase and cells were plated onto laminin-coated dishes in 1/4 DMEM F12 (Invitrogen) medium plus 3/4 Neurobasal (Invitrogen) supplemented with 5 mg/mL penicillin/streptomycin, 5 mg/mL L-Glutamine, 1% B27, 0.5% N2, 10 ng/ml FGF2 and 20 ng/ml BDNF (Peprotech, Tebu-Bio) for additional 3 days. Subsequently, for terminal differentiation, the cultures were exposed to 1/4 DMEM F12 medium plus 3/4 Neurobasal supplemented with 5 mg/mL penicillin/streptomycin, 5 mg/mL L-Glutamine, 1% B27, 0.5 % N2, 6.6 ng/ml FGF2 and 30 ng/ml BDNF and kept in these conditions for additional 7-14 days. During this period medium was changed every 3 days.

For neuronal differentiation of aNS-1 cells, the specific neuronal differentiation procedure was performed using 4 step-specific media which contain DMEM F12/NeuroBasal in specific ratios and definite supplements (D1-D4 media). The original detailed protocol is available elsewhere [15].

For adaptation to microplate format, both LC1 and aNS-1 cells were plated at a density of 30 × 10^3 ^cells/well in 96-well microplates and 16 × 10^3 ^cells/well in 384-well microplates. The medium changes required for the differentiation procedure were optimized so that only half of the media present in the well was removed and the same volume of fresh media was added. Fresh media for media change contained a double amount of growth factors in order to maintain the defined growth factors concentration, considering a complete growth factors depletion after 3 days in culture. Finally, to prevent problems related to media evaporation, the wells on the borders of the microplates were filled with Phosphate Buffered Saline (PBS) and not plated with cells (this means that 60 wells were considered useful for cell-plating in the 96-well microplates and 308 in the 384-well microplates).

### Immunofluorescence

At the indicated time points, proliferating or differentiated LC1 or aNS-1 cells were fixed by adding an equal volume of 8% formaldehyde to the media contained in the well. After 15 minutes at room temperature, cells were rinsed with PBS, permeabilized with 0.5% Triton X100-PBS for 15 minutes and incubated for one hour in blocking solution (5% fetal calf serum (FCS)-PBS or 2% BSA-PBS). Primary antibodies were diluted in blocking solution and applied overnight at 4°C. Primary antibodies used in this work are: anti beta III-tubulin (1:1000; Promega); anti BLBP (1:1000; Chemicon); anti cleaved caspase-3 (1:100; Cell Signaling); anti GFAP (1:750; DAKO); anti MAP2 (1:500; Chemicon); anti Nestin (1:250; Chemicon); anti Olig2 (1:500; Chemicon); anti Phospho Histone3 (1:100; Cell Signaling); anti Vimentin (1:100; Developmental Studies Hybridoma Bank). After three washes in PBS, appropriate secondary antibodies conjugated to Alexa fluorophores 488 or 568 (Molecular Probes, Invitrogen) were diluted at 1:500 in blocking solution in the presence of DAPI (Sigma Aldrich#D9564, 1 mg/ml) to counterstain the nuclei and applied for 2 hours at room temperature. The cells were then washed three times in PBS buffer.

### Images acquistion and analysis

The 20× images were acquired with a DMI3000 B microscope (Leica) or BD Pathway Bioimager 855 (Becton Dickinson) as indicated. Attovision software has been used for quantitative analysis.

### Cell proliferation assays

aNS-1 cells were plated in proliferation medium in 96- or 384-well microplates. cAMP-Glo Assay (Promega) or MTT viability assay (Sigma Aldrich) were performed starting one day after plating (0 h) every 24 hours (24 h, 48 h, 72 h).

### Apoptotic assays

Apoptotic assays have been optimized on proliferating LC1 cells and on differentiating aNS-1 cells. LC1 cells were plated in 96-well microplates at the density of 12 × 10^3 ^cells/well. The day after, NS expansion medium was replaced with the same medium containing the desired treatments. Caspase-Glo 3/7 Assay (Promega) was performed 48 hours after the exposure to the different treatments. Data were normalized using CellTiter-Blue Cell Viability Assay (Promega).

aNS-1 derived neuron like cells were exposed to differentiation medium containing the desired treatments_. _After 24 hours Caspase-Glo 3/7 Assay (Promega) was performed. Data were normalized using MTT Viability Assay.

### cAMP assay

aNS-1 derived neurons at the desired time after differentiation were exposed to the following treatments and concentrations in presence of phosphodiesterase inhibitors IBMX 100 mM and Ro20-1724 100 mM: SKF 38393 5 mM, 0.5 mM, 0,110 mM; quinpirole 100 mM, 10 mM, 1 mM; serotonine 100 mM, 10 mM, 1 mM, 0.1 mM, 0.01 mM, 0.001 mM; buspirone 100 mM, 10 mM, 1 mM, 0.1 mM, 0.01 mM, 0.001 mM; RS67506 100 mM, 10 mM, 1 mM, 0.1 mM, 0.01 mM; acetylcholine 100 mM, 10 mM, 1 mM, 0.1 mM, 0.01 mM, 0.001 mM. cAMP-Glo Assay (Promega) was then performed following the manufacturer's instructions.

### Becton Dickinson High-Content Image Analyses

aNS-1 cells were transiently transfected with plasmid pDS-YFP (H148Q, I152L, V163L) using the Mouse NSC Nucleofector Kit (Amaxa) according to the manufacturer's instructions and immediately plated in 96-well microplates in differentiation medium. Yellow fluorescent protein (YFP) is an engineered variant of green fluorescent protein, whose emission is quenched by small anions. The protein used is a further variant of YFP with three mutations, H148Q, I152L, V163S, each of which greatly enhances YFP Cl^- ^sensitivity [20]. Cells were differentiated for 21 days following the protocol previously described. Fluorescence expression levels were maintained at good levels during the differentiation procedure and were monitored with BD Pathway Bioimager 855 (Becton Dickinson) using a 20X objective (filter set for excitation at 500 ± 10 nm and emission 530 long pass emission filter). Immediately before the beginning of the experiment the culture media in each well was replaced with PBS (137 mM NaCl, 2.7 mM KCl, 0.7 mM CaCl_2_, 1.1 mM MgCl_2_, 1.5 mM KH_2_PO_4_, 8.1 mM Na_2_HPO_4_, pH7.4). Cells were exposed to increasing concentration of NaCl (ranging from 0 mM to 400 mM) to evaluate the effect of the extracellular Cl^- ^concentration on the emitted fluorescence.

GABA (10 μM, 1 mM, 20 mM) was dissolved directly in PBS and dispensed into each well using the injector equipped on the BD Pathway Bioimager 855. Images were acquired every 5 seconds after exposure to PBS only, NaCl or GABA. All experiments were performed at 37°C.

### Statistical analysis

Where indicated, data have been evaluated by ANOVA test.

## Results

### Adaptation of NS cells growth conditions to microplates

**Figure 1:**
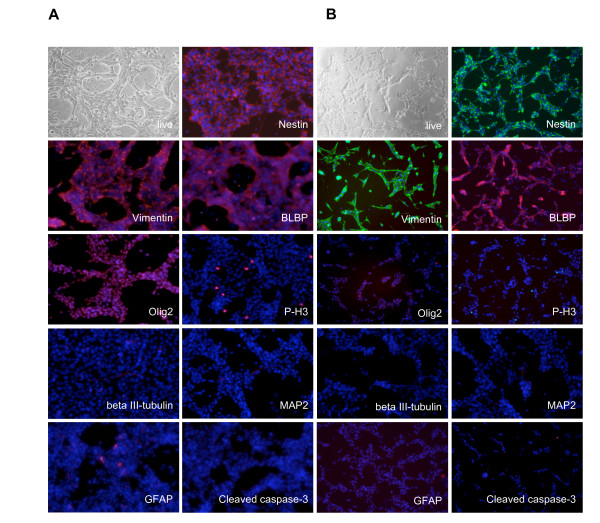
**Adaptation of culture conditions of aNS-1 cells to microplates**. aNS-1 cells can be cultured in 96- (**A**) and 384-well (**B**) plates, maintaining the expression of proper NS cells markers (Nestin, Vimentin, BLBP, Olig2), without exiting the cell cycle (Phospho-Histone3 immunoreactivity, P-H3), without differentiation (beta III-tubulin, MAP2 and GFAP absence) or cell death (cleaved caspase-3 absence).

Mouse NS cells are regularly grown in 25 cm^2 ^flasks and tested for their proliferation rate and differentiation capability in 24-well dishes. In these conditions, cells grow stably and homogenously while maintaining their pluripotency and neurogenic capacity [15,19]. In order to adapt their growth and differentiation potential to plate formats compatible with high-throughput screening, we have performed experiments in which seeding density and medium volume per well have been optimized to 96- and 384-well formats. After harvesting, aNS-1 cells, an adult SVZ-derived NS cell line [15], were plated at a density of 4 × 10^3^, 8 × 10^3 ^or 16 × 10^3 ^cells/well in 96-well and at a density of 1 × 10^3^, 2 × 10^3 ^or 4 × 10^3 ^cells/well in 384-well microplates. 80 μl and 45 μl respectively of growth medium containing FGF and EGF were added to the two formats. The day after plating and for the following three days, cells did not show any evident morphological change. After staining, they maintained expression of neural progenitor cell markers, such as Nestin, Vimentin, BLBP and Olig2 (Figure 1A and 1B). Analyses of Phospho Histone3 (P-H3) immunoreactivity shows that in both 96- and 384-well microplates cells undergo self-renewal in absence of unwanted differentiation as they do not express antigens proper of neurons such as beta III-tubulin and MAP2, nor of glial cells, such as GFAP (Figure 1A and 1B). Finally, the use of 96- or 384-well microplates did not affect the survival of the cells, as shown by the absence of active caspase-3 immunoreactivity (Figure 1A and 1B). Filling the wells on the border of the microplates with PBS, allowed the added media volume in each well to last for three days after plating. These growth conditions in 96- and 384-well formats have been successfully adapted also for LC1, a NS cell line derived from ESCs [13] (Additional file 1).

We conclude that mouse NS cells can be grown in microplates in the absence of differentiation or apoptosis.

### aNS-1 efficiently generate neurons in microplates

**Figure 2:**
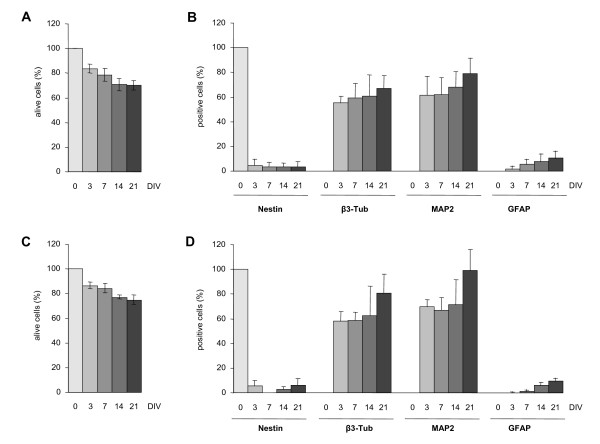
**aNS-1 microplates differentiation procedure, quantitative results**. (**A**) and (**C**): survival data during differentation in 96- (**A**) or 384- (**C**) well plates. (**B**) and (**D**): immunocytochemical marker expression in 96- (**B**) or 384- (**D**) well plates. The graphs show the relative proportion of alive cells expressing neural progenitor (Nestin), neuronal (beta III-tubulin, MAP2), and glial (GFAP) marker during differentation time. (**A**) and (**B**) show mean ± SD from seven different experiments in 96-well microplates. (**C**) and (**D**) show mean ± SD from 21 different replicates in the same experiment in 384-well microplates.

Our recently developed protocols for an efficient generation of mature neurons from adult SVZ-derived and ESCs-derived NS cells [16,20] were adapted to microplates. We started by setting up parameters such as seeding density and medium volume per well and absence of evaporation. As we noticed that replacing the entire medium was not appropriate for the cells we adopted a partial medium change during differentiation We firstly focused on aNS-1 cells and evaluated their survival during the differentiation procedure in the different microplate formats. 40 × 10^3^, 30 × 10^3 ^and 20 × 10^3 ^cells/well were plated in 96-well microplates and 16 × 10^3 ^and 10 × 10^3 ^cells/well in 384-well microplates, directly in 100 μl of D1 differentiation medium and analyzed after 3, 7, 14 or 21 days *in vitro *(DIV). At each time point, 8 or 21 single wells from a 96- or 384-well microplate, respectively, were fixed and nuclei stained with DAPI. In order to obtain quantitative and objective results, we used the BD Pathway Bioimager 855 and the Attovision software. For each well, 16 fields chosen through a geometric scheme independent from the operator, have been scanned via the BD Pathway. The single color images were then analyzed via the Attovision software, by using masks specifically designed to discriminate signal intensity and signal shape, in order to select functional nuclei and small picnotic nuclei. The number of alive cells was then obtained by subtracting the number of picnotic cells from the total cell number. An exemplificative picture of the masks produced by the Attovision software to obtain the indicated data is presented in Additional file 2. For each condition, an average of 4 × 10^3 ^cells/well were counted in the 96-well. Average was 10 × 10^3 ^cells/well for 384-wells. Six independent experiments have been carried out in 96-wells and a single one in 384-well format. Importantly, we found that after 21 days of differentiation, which is the best timing for aNS-1 neuronal differentiation [15], 75% of the initially plated cells were still alive, both in the 96- and 384-well format (Figure 2A and 2C, respectively; see Table 1 for detailed results). We subsequently analyzed the maturation of the cells in culture, by assessing the presence of specific antigens at day 3, 7, 14 and 21. For this aim, an automated counting procedure was adopted in a multiplexing acquisition mode using a mask specifically designed on the peculiar shape of differentiated aNS-1 cells. Three color-images have been acquired from each selected field (DAPI to stain nuclei, Alexa Fluor 488 for monoclonal Nestin and beta III-tubulin, Alexa Fluor 546 for polyclonal GFAP and MAP2) (Additional file 3) and analyzed via the Attovision software. An average of 1700 and 700 cells were counted in 96-well and in 384-well microplates, respectively. As expected, three days after the beginning of the differentiation procedure, the expression of Nestin was found reduced to about 5% and is maintained in this range during the entire differentiation procedure, with the same values being observed in 96- and in 384-well microplates (Figure 2B and 2D). The immunocytochemical analysis with antibodies against beta III-tubulin and MAP2 shows the expected increase in the number of immunoreactive cells with the majority of them expressing beta III-tubulin and MAP2 at day 21 in both microplate formats (Figure 2B and 2D). Finally, as shown by GFAP immunoreactivity and subsequent analysis, the percentage of glial cells in these cultures is very low (about 3-5%) (Figure 2B and 2D). Surprisingly, this percentage obtained both in 96-well and 384-well microplates is even lower than what originally observed in the 24-well format [13,15]. The differentiation procedure performed in microplates shows a strong well to well reproducibility, as assessed by beta III-tubulin and MAP2 expression in six independent wells from a 96-well microplate (Additional file 4). Percentage of immunoreactive cells are also kept homogeneous among the different wells at all time points (3, 7, 14 and 21 days of differentiation). We also analyzed BrdU incorporation along differentiation, confirming that NS cells gradually stop proliferating during differentiation (DIV1: 28.39% ± 2.71, DIV7: 6.87 ± 0.85, DIV14: 0.79% ± 0.15). The best results in terms of cell distribution in the well that led to a density of differentiated aNS-1 cells suitable for automated microscopy and image analysis from 3 to 21 days after plating, were obtained when plating 30 × 10^3 ^cells/well in 96-well and 16 × 10^3 ^cells/well in 384-well microplates. At the time of analysis, nuclei were well separated from each other and cells were distributed evenly over the area of each well. Moreover, in this condition, only a few overlapping nuclei are present, and we were able to exclude them in the image analysis process thanks to specifically designed masks. We also noticed that a higher cell density is needed in these formats with respect to the number of cells per well optimized for 24 well plates (i.e. 50 × 10^3 ^cells/cm^2 ^in 24-well plates vs 93 × 10^3 ^cells/cm^2 ^in 96- and 145 × 10^3 ^cells/cm^2 ^in 384-well microplates). Under these conditions, neuronal differentiation of NS cells in microplates is robust and reproducible, as evident from Table 2 in which 7 independent differentiation experiments performed on aNS-1 cells at different *in vitro *passages are presented. Moreover, in Table 3 it is possible to appreciate a direct comparison between the differentiation efficiency in 96- and 384-well microplates that highlights the reproducibility of our differentiation procedure in different plate formats. These data are in agreement with the results obtained with the differentiation of aNS-1 in standard 24-well plate format [15]. We conclude that we have successfully adapted the differentiation of aNS-1 to microplates.

**Table 1:** aNS-1 cell culture survival during differentiation in 96-well microplates

	Exp #	Passage #	Alive cell (%)
DIV3	1 2 2' 4 5 7	18 23 23 32 32 39	81.92 ± 2.86 (n = 3783) 81.29 ± 4.55 (n = 4017) 86.38 ± 2.58 (n = 10425) n.a. n.a. 87.20 ± 1.71 (n = 5158)
			
DIV7	1 2 2' 4 5 7	18 23 23 32 32 39	75.61 ± 2.80 (n = 5166) 74.01 ± 1.05 (n = 4394) 84.28 ± 3.74 (n = 11681) 76.40 ± 2.12 (n = 4457) 81.82 ± 1.38 (n = 4531) 85.70 ± 2.40 (n = 7310)
			
DIV14	1 2 2' 4 5 7	18 23 23 32 32 39	75.41 ± 3.16 (n = 4000) 66.72 ± 11.79 (n = 3595) 76.67 ± 2.69 (n = 11441) 74.96 ± 1.43 (n = 3757) 71.51 ± 2.32 (n = 3526) 65.01 ± 3.28 (n = 4947)
			
DIV21	1 2 2' 4 5 7	18 23 23 32 32 39	66.81 ± 2.92 (n = 3423) 74.77 ± 5.32 (n = 3988) 74.91 ± 3.72 (n = 7433) 72.44 ± 3.47 (n = 4187) 66.55 ± 6.19 (n = 3503) 69.66 ± 3.38 (n = 5108)

Data were evaluated by Attovision software. Experiments 1, 2, 4, 5, 7 were performed in 96-well microplates. Experiment 2' was performed in 384-well microplate. Experiment 2 and 2' were run in parallel. aNS-1 cells were maintained *in vitro *for the indicated number of passages (Passage #); n: number of cells analyzed.

**Table 2:** Relative proportion of aNS-1 cells expressing neural progenitor, neuronal and glial antigens during differentiation in 96-well microplates

	Exp #	Passage #	Nestin	beta III-tubulin	MAP2	GFAP
DIV3	1 2 3 4 5 6 7	18 23 25 32 32 33 39	0 (n = 3074) 6.06 ± 3.34 (n = 608) n.a. n.a. n.a. 10.00 ± 1.60 1.90 ± 0.52 (n = 1805)	52.22 ± 3.10 (n = 3134) 57.12 ± 14.03 (n = 661) n.a. n.a. n.a. 51.47 ± 10.63 60.93 ± 0.63 (n = 2990)	64.13 ± 9.63 (n = 3074) 63.80 ± 8.12 (n = 608) n.a. n.a. n.a. 45.60 ± 17.26 74.35 ± 4.71 (n = 1805)	0.17 ± 0.08 (n = 3134) 0.55 ± 0.61 (n = 661) n.a. n.a. n.a. 5.30 ± 2.55 0.09 ± 0.15 (n = 2990)
						
DIV7	1 2 3 4 5 6 7	18 23 25 32 32 33 39	0.4 ± 0.07 (n = 4711) 1.36 ± 1.05 (n = 659) 3.14 ± 1.61 8.58 ± 3.72 (n = 366) 4.50 ± 2.02 (n = 367) n.a. 1.64 ± 0.37 (n = 3791)	54.26 ± 1.59 (n = 3639) 59.91 ± 16.55 (n = 695) 40.27 ± 6.79 65.40 ± 14.70 (n = 470) 69.58 ± 10.74 (n = 453) n.a. 66.86 ± 3.97 (n = 3666)	61.06 ± 7.56 (n = 4711) 61.31 ± 7.59 (n = 659) 39.02 ± 1.62 72.62 ± 9.71 (n = 366) 63.98 ± 12.95 (n = 367) n.a. 74.89 ± 1.68 (n = 3791)	6.61 ± 2.58 (n = 4711) 2.13 ± 1.36 (n = 695) 5.33 ± 2.19 6.81 ± 8.91 (n = 470) 11.96 ± 4.46 (n = 453) n.a. 0.43 ± 0.23 (n = 3666)
						
DIV14	1 2 3 4 5 6 7	18 23 25 32 32 33 39	0.40 ± 0.28 (n = 4475) 30.36 ± 3.61 (n = 715) 2.75 ± 2.15 7.39 ± 4.36 (n = 361) 4.57 ± 2.81 (n = 428) 3.49 ± 1.67 0.61 ± 0.37 (n = 2383)	40.94 ± 4.28 (n = 3416) 59.45 ± 9.59 (n = 636) 57.45 ± 7.19 66.05 ± 20.84 (n = 346) 77.40 ± 15.95 (n = 413) 43.52 ± 4.19 82.57 ± 3.86 (n = 2584)	74.25 ± 17.00 (n = 4475) 87.29 ± 6.77 (n = 715) 61.35 ± 7.16 61.67 ± 12.06 (n = 361) 62.67 ± 13.52 (n = 428) 52.72 ± 2.42 75.35 ± 5.32 (n = 2383)	4.43 ± 1.82 (n = 3416) 4.94 ± 1.48 (n = 636) 13.45 ± 4.13 8.70 ± 5.87 (n = 346) 17.60 ± 8.73 (n = 413) 1.94 ± 1.86 4.63 ± 0.21 (n = 2584)
						
DIV21	1 2 3 4 5 6 7	18 23 25 32 32 33 39	0 (n = 2968) 5.36 ± 3.40 (n = 598) 3.00 ± 1.33 6.35 ± 3.13 (n = 457) 6.13 ± 3.86 (n = 466) n.a. 0.6 ± 0.39 (n = 2537)	62.29 ± 3.23 (n = 2455) 68.38 ± 11.07 (n = 573) 56.72 ± 6.65 59.95 ± 11.36 (n = 381) 75.10 ± 13.99 (n = 395) n.a. 79.89 ± 16.80 (n = 2571)	92.60 ± 7.59 (n = 2968) 90.80 ± 14.67 (n = 598) 70.04 ± 5.16 68.11 ± 17.83 (n = 457) 84.65 ± 14.04 (n = 466) n.a. 68.90 ± 10.77 (n = 2537)	5.85 ± 0.25 (n = 2455) 7.70 ± 3.29 (n = 573) 5.10 ± 0.73 16.60 ± 5.08 (n = 381) 18.20 ± 7.02 (n = 395) n.a. 10.33 ± 3.19 (n = 2571)

Data were evaluated by Attovision software analysing aNS-1 alive cells expressing neuronal (beta III-tubulin, MAP2), neural progenitor (Nestin) and glial (GFAP) markers. Results were obtained from seven independent experiments performed by starting with aNS-1 cells maintained *in vitro *for the indicated number of passages are shown. Each experiment was performed in 96-well microplates; plating density was 30 × 10^3 ^cells/well; n: number of cells analyzed.

**Table 3:** Relative proportion of aNS-1 cells expressing neural progenitor, neuronal and glial antigens during differentiation in 96- and 384-well microplates

	Plate format	Nestin	beta III-tubulin	MAP2	GFAP
DIV3	96W 384W	6.06 ± 3.34 (n = 608) 5.51 ± 3.45 (n = 155)	57.12 ± 14.03 (n = 661) 58.16 ± 7.14 (n = 904)	63.80 ± 8.12 (n = 608) 69.49 ± 5.07 (n = 882)	0.55 ± 30.61 (n = 661) 0.10 ± 0.30 (n = 904)
					
DIV7	96W 384W	1.36 ± 1.05 (n = 659) n.a.	59.91 ± 16.55 (n = 695) 58.35 ± 6.20 (n = 999)	61.31 ± 7.59 (n = 659) 66.82 ± 9.47 (n = 738)	2.13 ± 1.36 (n = 695) 1.30 ± 1.04 (n = 999)
					
DIV14	96W 384W	3.36 ± 3.61 (n = 715) 2.84 ± 1.05 (n = 788)	59.45 ± 9.59 (n = 636) 62.54 ± 22.95 (n = 763)	87.29 ± 6.77 (n = 715) 71.53 ±19.17 (n = 788)	4.94 ± 1.48 (n = 636) 5.82 ± 2.65 (n = 763)
					
DIV21	96W 384W	5.36 ± 3.40 (n = 598) 6.00 ± 4.76 (n = 447)	68.38 ± 11.07 (n = 573) 80.89 ± 14.57 (n = 484)	90.80 ± 14.67 (n = 598) 98.76 ± 16.00 (n = 333)	7.70 ± 3.29 (n = 573) 9.22 ± 2.49 (n = 484)

Data were evaluated by Attovision software analysing aNS-1 alive cells expressing neuronal (beta III-tubulin, MAP2), neural progenitor (Nestin) and glial (GFAP) markers; 96- and 384-well microplates experiments were run in parallel; n: number of cells analyzed.

We next applied a similar process for LC1 cells, adjusting to microplates the specific differentiation procedure of ESCs-derived NS cells, which differs from the protocol used for aNS-1 cells because no replating of the predifferentiated cells is necessary [19]. In this case also, 30 × 10^3 ^cells were plated in 96-well and 16 × 10^3 ^cells in 384-well plates and a partial medium change was applied during the differentiation process. The images were acquired following the same stochastic parameters used for aNS-1 cells and optimized masks for differentiated LC1 cells morphology were developed. Again results showed the expected decrease in Nestin immunoreactivity, paralleled by an increase in beta III-tubulin and MAP2 positive cells along the differentiation. GFAP immunoreactivity was present at very low levels at each analyzed time point (Additional file 5 and data not shown).

So far, these results indicate that mature neurons obtained from both aNS-1 and LC1 NS cells are suitable for High-Content assays in a High-Throughput format.

### Development of cell proliferation assays based on NS cells

**Figure 3:**
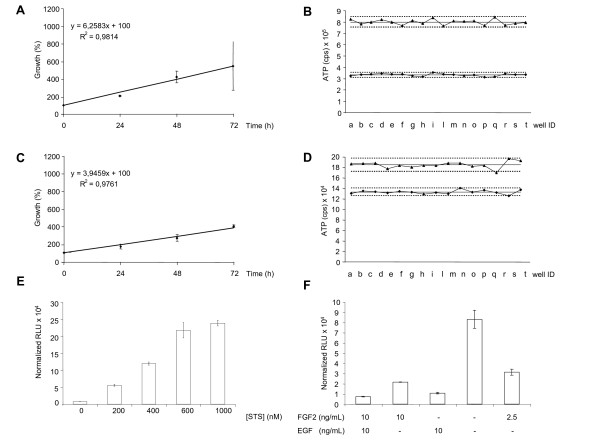
**Cell proliferation and cell death assays**. (**A**) and (**C**): Graphs of representative linear correlations in ATP assay performed in aNS-1 proliferating cells. 8 × 10^3 ^cells/well plated in 96-well microplates (**A**) and 4 × 10^3 ^cells/well plated in 384-well microplates (**C**); data are expressed as growth (%), referred to the value obtained at t = 0. The data are the mean ± SD from three independent experiments, each one performed in triplicate. (**B**) and (**D**): Data dispersion around mean value (full line) from ATP assays performed in 18 wells from a 96- (**B**) or a 384- (**D**) well microplate; upper and lower groups represent 48 h and 24 h data respectively; letters in x axes identify each individual well; dot lines represent 2SD from mean values. (**E)**: Activation of Caspases 3/7 in LC1 cells exposed to the indicated doses of staurosporine for 6 hours. Data are normalized on cell viability, as assessed by CellTiter-Blue Cell Viability Assay (**F)**: Activation of Caspases 3/7 in LC1 cells exposed to the indicated stress conditions for 48 hours. Data are normalized on cell viability. RLU: Relative Luciferase Unit.

Based on the above evidence we set out to test NS cells in high-content assays to measure their growth curve and doubling time in 96- and 384-well microplates. We first set the conditions for application of an ATP assay (CellTiter Glo Luminescent Cell Viability Assay, Promega) on aNS-1 cells. A first series of experiments was designed to test the impact on the proliferation rate of different seeding densities of aNS-1 cells. We found that for 96-well microplates the best condition was 8 × 10^3 ^cells/well; at this seeding density the doubling time of NS cells is about 31 h (Figure 3A), as previously reported by our group [13] and the analysis of the correlation coefficient revealed a R^2 ^= 0.9814. These results represent the average data obtained from three independent experiments. The intraplate reproducibility is also appropriate, with a Coefficient of Variation (CV) of 0.0334 at 24 h and 0.0294 at 48 h when calculated among 18 wells (Figure 3B). In 384-well microplates, the best condition for an ATP assay is 4 × 10^3 ^cells/well, with a doubling time of 34 h (Figure 3C; correlation coefficient is R^2 ^= 0.9761 as calculated from three independent experiments). The CV calculated among 18 wells is 0.0270 at 24 h and 0.0338 at 48 h. For results obtained at the other tested densities, we invite the reader to refer to Additional file 6.

We next tried to set up the MTT assay as an alternative assay for cell proliferation [21]. MTT assay applied to NS cells in microplates presents a higher intraplate variability (CV = 0.1977 after 24 h and CV = 0.0922 after 48 h of proliferation in 96-well formats) and a lower correlation coefficient (R^2 ^= 0.9301) with respect to the ATP assay. We therefore elected the ATP measurement as the method of choice to count alive proliferating NS cells in microplates. Reproducible and robust results have been obtained also by plating 12 × 10^3 ^LC1 cells in 96-well and 4 × 10^3 ^cells in 384-well formats (data not shown).

### Application of cell death assays to self-renewing NS cells

The Caspase-Glo 3/7 Assay (Promega) is a homogeneous, luminescent assay that measures the activities of caspase-3 and -7 enzymes, [22-24]. We used this assay to monitor apoptotic cell death in proliferating LC1 cells. Therefore LC1 cells were first plated in 96-well microplates and exposed to different doses of staurosporine, a well known proapoptotic molecule, ranging from 200 to 1000 nM. After six hours of exposure to this protein kinase inhibitor, we performed the Caspase-Glo 3/7 Assay. As shown in Figure 3E, exposure of NS cells to staurosporine causes a dose-dependent increase in luciferase signal, paralleling the activation of caspases.

We subsequently tested the activation of caspases in NS cells exposed to stress conditions. NS cells are isolated and routinely expanded in the presence of EGF and FGF2 [13]. Once a NS cell line is established, it is possible to remove FGF2 and expand NS cells in the presence of EGF only [14]. In fact, although FGF2 is specifically required for initial derivation of NS cells, addition of EGF is important for expansion of homogenous NS cell cultures with efficient suppression of differentiation and apoptosis. We plated LC1 cells in 96-well plates in five different conditions, as described in Figure 3F. After 48 hours, we performed the Caspase-Glo 3/7 Assay. As shown in Figure 3F, removal of both growth factors causes a dramatic activation of caspases reaching 83 × 10^3 ^normalized RLU (Relative Luciferase Units) that are reduced to 31 × 10^3 ^normalized RLU in the presence of 25% of the regular dose of FGF2. The absence of EGF induces the activation of these enzymes, but at much lower levels if compared to the removal of both growth factors (respectively 21 × 10^3 ^versus 83 × 10^3 ^normalized RLU). Finally, the absence of FGF2 does not evoke any increase in caspase activity; in fact the level of caspases activation is similar to the value obtained in standard culture conditions (11 × 10^3 ^versus 7 × 10^3 ^normalized RLU).

In conclusion, we have shown that it is possible to measure the activation of specific apoptotic enzymes, such as caspase-3 and -7, in NS cells plated in 96-well microplates and exposed to a number of chemical insults such as staurosporine or growth factor withdrawal, indicating that proliferating LC1 cells might be a useful tool in the study of chemicals or molecules impacting the apoptotic machinery.

### aNS-1-derived neurons to investigate oxidative stress

**Figure 4:**
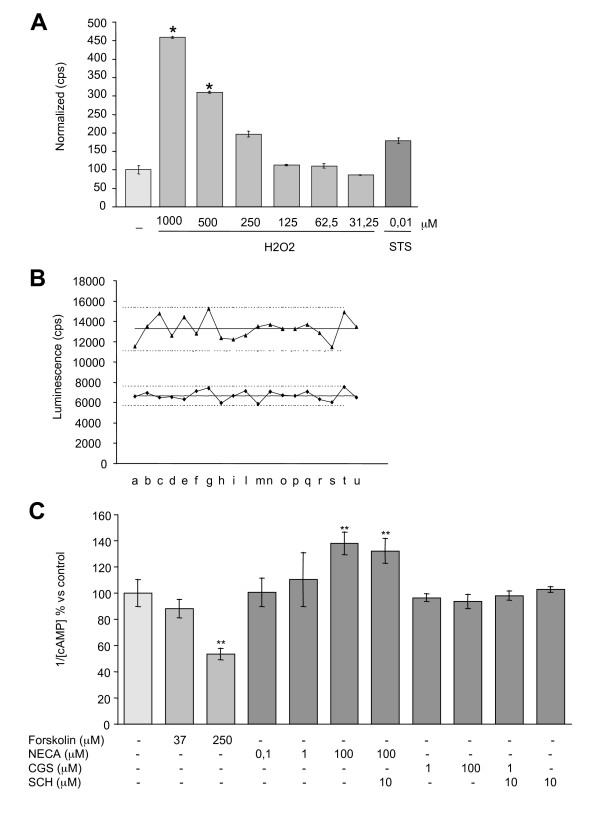
**Homogeneous assays in aNS-1 derived neurons**. (**A**): 3DIV aNS-1 differentiated cells were exposed to H_2_O_2 _or STS in 96-well microplates for 24 h. Caspase 3/7 activation, normalized on vitality data as assessed by MTT assay, is reported. Results from one experiment performed in triplicate are shown. Three independent experiments give rise to overlapping results. (**B**): 21DIV aNS-1 differentiated cells were exposed to Forskolin 250 μM for 15' and cAMP-Glo Assay™ was then performed on 19 well from a 96-well microplate. Row data dispersion around mean value (full line) is reported; upper and lower groups represent untreated control and treated well data respectively; dot lines represent 2SD from mean values: letters in x axes identify each individual well. (**C**): 21DIV aNS-1 differentiated cells were exposed to adenosine receptor agonist/antagonist for 1 h. Forskolin for 15' was used as positive control. Graph shows cAMP levels modulation, average value from three independent experiments performed in triplicate. * p < 0.05, ** p < 0.01. H_2_O_2_: Hydrogen Peroxide; cps, counts per second; STS: staurosporine.

Oxidative stress has long been linked to neuronal cell death that is associated with certain neurodegenerative conditions. Neurons are particularly prone to oxidative stress and inadequately equipped with antioxidant defense systems to prevent "ongoing" oxidative damage [25]. As we moved to the optimization of homogenous assays on neuronally differentiated NS cells we have optimized the use of aNS-1 derived neurons as a tool for the detection of hydrogen peroxide induced apoptosis. aNS-1 cells were plated in 96-well microplates and exposed to the differentiation conditions. After three days, cells were exposed to different doses of H_2_O_2 _(from 31.25 to 1000 mM) or staurosporine 0.01 mM as positive control and the Caspase-Glo 3/7 Assay (Promega) was performed at the indicated time points. Data were normalized on viability data as assessed by MTT assay, which is more sensitive than ATP assay on differentiated aNS1-cells (data not shown). We observed that, as expected, after 6 h of exposure, staurosporine induces a statistically significant activation of caspases (data not shown). After 24 h, 1 mM and 500 μM H_2_O_2 _cause a statistically significant and dose-dependent activation of caspases (Figure 4A). Furthermore, while the control group showed the expected neuronal morphology and dendritic networks, incubation with 250 mM, 500 mM or 1 M H_2_O_2 _for 24 h caused a proportional decrease in the number of viable cells (data not shown).

### aNS-1 as a tool for screening of drugs/molecules able to modulate cAMP levels

Neurons derived from aNS-1 cells have been tested for their suitability in the evaluation of G-protein coupled receptors (GCPRs) activity. The cAMP-Glo Assay (Promega) is a homogenous, non-radioactive assay, which we have optimized for the use on differentiated aNS-1 cells in 96-well microplates. In these experiments, Forskolin, a cell-permeable direct activator of Adenylate cyclase, was taken as a reference compound for assay validation. Exposure of aNS-1 cells to Forskolin 250 mM for 15 minutes at day 21 of differentiation, gives rise to the expected statistically significant increase in cAMP production (- 45% of luminescence signal, Figure 4B). The intraplate dispersion in the 96-well microplates was assessed in 19 separate wells treated with Forskolin 250 mM versus 19 untreated wells. In both conditions the observed spread of the data is satisfactory for the purpose (Figure 4B). Moreover, the assay has been validated on aNS-1 cells differentiated for 3, 7 and 14 days and the results overlap with those obtained at day 21 (- 35% at 3 days, - 35% at 7 days and - 40% at 14 days with respect to untreated samples).

Subsequently, at day 21, aNS-1 cells have been exposed to a panel of known agonists and antagonists of GPCRs coupled receptor. Considering the Adenosine receptor system, three agonists/antagonists have been assessed *per se *or in combination: namely NECA (A1, A2a, A3 agonist; 0.1 mM, 1 mM, 100 mM), CGS21680 (A2a agonist; 1 mM, 100 mM) and SCH58261 (A2a antagonist; 10 mM).

aNS-1 cell cultures differentiated for 21 days and once exposed to NECA 100 mM showed a statistically significant decrease of cAMP levels (+ 40% with respect to untreated samples, Figure 4C). Using the adenosine A2a agonist CGS21680 or the adenosine A2a antagonist SCH58261 in combination with NECA, we did not observe variations in NECA mediated cAMP modulation (Figure 4C). These observations led us to conclude that A1/A3 adenosine receptors, but not A2a receptor, are present and functional in aNS-1 cells which were differentiated for 21 days. Similar results were obtained after 3, 7 and 14 days of differentiation. Under the same experimental conditions we have also tested SKF 38393 (a D1 like agonist), quinpirole (a D2 like agonist), serotonine and buspirone (5-HT_1A _agonists), RS67506 (a 5-HT_4 _agonist) and acetylcholine for their ability to influence GPCRs. However, none of these stimuli at the doses indicated in the method section gave rise to a statistically significant modulation of cAMP levels (data not shown).

### aNS-1-derived YFP* reporter neurons as a tool to screen for molecules active on GABA-A receptor

**Figure 5:**
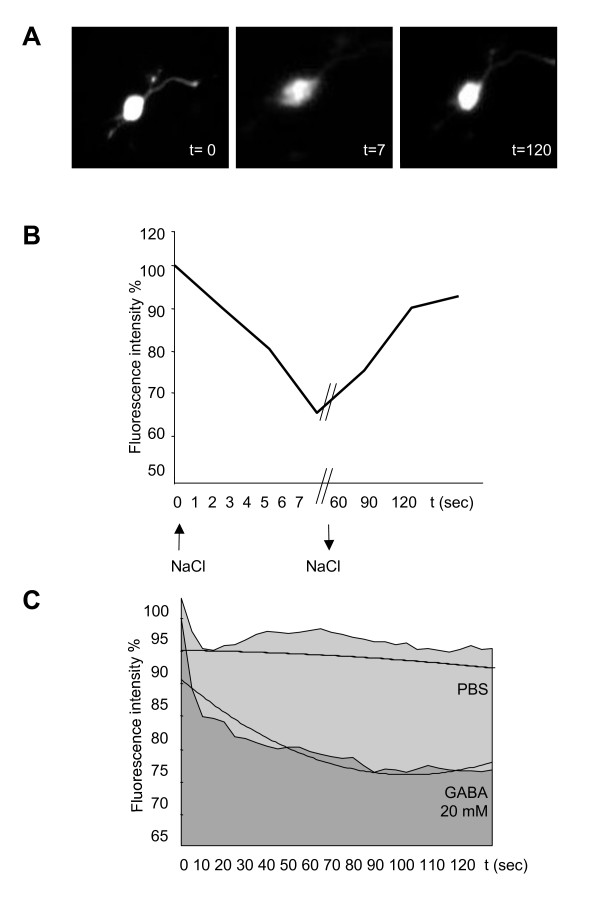
**An YFP based assay for the measurement of GABAA receptor activity**. (**A**) and (**B**): Extracellular Cl- overload produces a rapid decrease in cellular fluorescence that reaches the minimum in 7 sec. The fluorescence decrease is reversible, with complete recovery of resting fluorescence occurring within 2 minutes after removal of extracellular Cl-. (**C**): 21DIV aNS-1 YFP* cells have been exposed to GABA 20 mM or PBS only as a control. Fluorescence emission intensity has been recorded via AttoVision (BD Bioscience) every 5 seconds. Data elaboration through the integration of the area under the curve shows a decrease of the fluorescence signals specifically in the cells exposed to GABA.

We next tested the validity of NS-derived neurons in High-Content functional assays. In particular, we focused on the evaluation of the GABA-A receptor, previously shown to be expressed and functionally active in neurons derived from aNS-1 cells [15]. In order to evaluate the activity of this receptor, we have used a mutant form of the YFP protein (YFP*), which is particularly sensitive to anion levels variations [20]. Proliferating aNS-1 cells were transiently transfected through the nucleofection method (Amaxa) with the YFP* construct and then exposed to differentiation procedure. After 21 days of differentiation in 96-well microplates, expression of the exogenous fluorescent protein was still present (Figure 5A, t = 0). After exposure of 21DIV aNS-1 cells to 20 mM NaCl, a rapid decrease in the fluorescence signal was observed (- 60% fluorescence intensity with respect to PBS only treated cells, Figure 5A, t = 7 sec; Figure 5B), that was restored 2 minutes after replacing the physiological Chloride concentration (Figure 5A, t = 120; Figure 5B), thus demonstrating YFP* Chloride sensitivity in aNS-1 cells. We also show that differentiated NS-YFP* cells are sensitive to GABA stimuli. In fact, a decrease in the fluorescence signal is detected starting 10 seconds after exposure to GABA 1 mM in PBS. To obtain quantitative results, images of the treated cells were acquired by the BD Pathway Bioimager 855 (Becton Dickinson) every 5 seconds after exposure to GABA or PBS only for a 30 minutes period. The acquired images have then been analyzed by ImageJ software in order to obtain the fluorescent signal intensity for each positive cell over time. These data (fluorescent signal intensity versus time of exposure to the stimuli) have been reported in graph, and the area under the curve (AUC) has been used as a parameter referred to the response to GABA stimuli (Figure 5C). We report that the average AUC measured for the 14 PBS treated cells analyzed was 2660 ± 108, while on 13 cells exposed to 1 mM GABA the average AUC was 2399 ± 105, indicating a statistically significant decrease in the fluorescent signal specifically due to GABA exposure (- 9.81%). This evidence reinforces previous data showing GABA responsiveness in NS derived neurons [15].

## Discussion

The development of reliable and reproducible cell-based assays is a major goal in the current panorama of drug screening where the application of cellular systems highly resembling specific physiological contexts is strongly required. The obtainment of a large amount of specialized cells is, however, not trivial when thinking of subtype specific, non-proliferating cellular populations. The potential offered by stem cells in this sector appears enormous thanks to their capability to self renew *in vitro *and to produce interesting specialized phenotypes. Stem cells may therefore be suitable for drug screening application, in particular for the discovery of CNS-active compounds, where primary cultures are more difficult to obtain in the amounts required by HTS settings.

Here we report the adaptation of homogenous assays and the development of High-Content assays in mouse NS cells, an homogenous population of tissue-specific stem cells that can be long-term propagated in the absence of unwanted differentiation [13,14]. We thus demonstrate that NS cells can be maintained in active proliferation in 96- and 384-wells microplates and can be adapted to homogeneous assays for the discovery of molecules capable of activating and mobilizing endogenous NS cells as well as for the identification of new genes involved in the maintenance of the self renewal state or in the differentiation processes. Being the procedure to derive NS cells well defined [13,14], there is the possibility to extend the application of the herein validated homogeneous assays on NS cells obtained from mice carrying genetic mutation of interest. Moreover it is also appealing to note that other neural stem cell systems have been validated for their capability to highlight the activity of compounds of interest. For example, in the work by Chiou et al. [26] Prozac was demonstrated able to increase the cell viability and proliferation of hippocampal derived neural stem cells and to inhibit the production of proinflammatory cytokines [26]. Validated and homogeneous neural stem cell systems are also needed as a basis for RNAi screens that select for proteins that play crucial roles in stem cell behavior. Furthermore, a specific interest in the availability of homogeneous neural stem cell systems arises from the hypothesis that brain tumors are maintained by rare cancer cells with stem cell-like properties [27].

Here we have adapted our optimized differentiation protocol for NS cells [15] to 96- and 384-wells microplates and developed homogeneous or High-Content screenings through BD Pathway Bioimager 855 for the evaluation of cell viability and differentiation efficiency using a protocol that recapitulates different steps of neuronal maturation. The automated analysis of several differentiation trials also demonstrates that our microtiter differentiation protocol is reproducible, robust and efficient. The obtained differentiated cultures are rather homogeneous for the expression of neuronal markers, and the homogeneity of the culture is mandatory in a HTS setting.

Experiments performed on aNS-1-derived YFP* reporter neurons indicate that exogenous genes are maintained during the differentiation procedure, suggesting that the engineering approach with a receptor of interest is feasible. This is of particular interest given that currently, except for primary cells, there is no other neuronal cell line available to monitor functionally active neuronal-specific receptors. For example, although electrophysiological studies have shown the presence of currents elicited by glutamate agonists in neuronally differentiated NS cells [15], the same cells are not sensitive to glutamate receptor activation. In fact, aNS-1 cells differentiated *in vitro *for 21 days and exposed to glutamate at the concentrations currently used in primary cortical neurons to elicit cell death (ranging from 5 μM to 10 mM) did not undergo cell death, as revealed by LDH and ATP assay (data not shown). The engineering approach pursued in NS cells may carry in the advantage of a correct neuronal context in which to study the receptor of interest, which is not present in the widely used engineered immortalized cells,. Furthermore, the possibility to use engineered NS cells differentiated in microplate format allows the development of reporter based assays useful to monitor a specific cell fate. For example, a random activation gene expression (RAGE) approach [28] could be an option for the discovery of new genes involved in the obtainment of a specific neuronal population [29] and Albieri I, Onorati M, Calabrese G, Moiana A, Badaloni A, Camnasio S, Spiliotopoulos D, Cattaneo E, Consalez. GG: A DNA-transposon-based approach to functional screening in Neural Stem cells, submitted].

## Conclusions

A thorough characterization of the available neural stem cell systems is mandatory to understand their limits and potential applications. Neurons from NS cells do not replicate the full repertoire of neurochemical attributes and functions that are present in primary neurons. Yet, they offer the great advantage of being derived from stable and replicating sources of stem cells which can be maintained in monolayer and serum free conditions, grown and efficiently differentiated in microtiter plates. These properties make them suitable for High Content and High throughput screening approaches searching for compounds and new genes active in normal and diseased neurons.

## Authors' contributions

DR, EC have designed the study; AG and DB carried out aNS-1 cells differentiation and performed immunoassays and cell based assay on them. AM carried out LC1 cells differentiation and performed immunoassays and cell based assay on them. SC and RP performed experiments with aNS-1-derived YFP* reporter. AG, AM, DR, EC have reviewed the data; AG, SC, DR, EC have written the manuscript.

All authors read and approved the final manuscript.

## Supplementary Material

Additional file 1 **Adaptation of culture conditions of LC1 cells to microplates. **LC1 cells can be cultured in 96-well plates, maintaining the correct expression of NS cells markers (Nestin, Vimentin, BLBP, Olig2, Phospho-Histone3), without differentiation (beta III-tubulin, MAP2 and GFAP absence) or cell death (cleaved caspase-3 absence). Click here for file

Additional file 2 **Examples of the masks designed by the Attovision software. **The single colour images, acquired by the BD pathway, were analysed via Attovision software by using specifically designed masks. **(A) **Example of the mask designed to count DAPI positive alive cells. **(B) **Mask created to count the total number of cells. **(C) **Mask able to select picnotic cells only. Click here for file

Additional file 3 **Differentiating aNS-1 cells progressively acquire neuronal antigenic properties when plated in microplates**. Downregulation of NS cells markers such as Nestin (green) and upregulation of neuronal markers beta III-tubulin (green) and MAP2 (red) in aNS-1 cells between DIV3 and DIV21. Low levels of glial marker GFAP (red) were observed at all DIV times. Nuclei were stained with DAPI (blue; all panels). Click here for file

Additional file 4 **Intra experiment reproducibility of aNS-1 differentiation procedure**. Graphs show relative proportion of alive cells expressing neuronal markers in 6 independent wells from a same 96-well microplate. (**A**): beta III-tubulin expression. (**B**): MAP2 expression. Full lines represent mean values. Click here for file

Additional file 5 **Differentiation of LC1 cells in 96-well plates**. Immunofluorescence experiment showing the expression of markers during the differentiation of LC1 cells in 96-well plates. Most of the differentiating NS cells lose Nestin expression, acquiring a neuronal phenotype (beta III-tubulin and MAP2 immunoreactivity) instead of becoming glial cells (GFAP immunoreactivity). Click here for file

Additional file 6 **Cell proliferation assays in 96- and 384-well microplates**. **(A)**and **(C)**Graphs of representative linear correlations in ATP assay performed in aNS-1 proliferating cells, 4 × 10^3 ^cells/well **(A) **and 16 × 10^3 ^cells/well **(C) **plated in 96-well microplates; (**A**) and (**C**): data are expressed as growth (%), referred to the value obtained at t = 0. The data are the mean ± SD from three independent experiments, each one performed in triplicate. (**B**) and (**D**): Data dispersion around mean value (full line) from ATP assays performed in 18 wells from 1 × 10^3 ^**(B) **or 2 × 10^3 ^(**D**) cell/well plated in 384-well microplates; upper and lower groups represent 48 h and 24 h data respectively; letters in x axes identify each individual well; dot lines represent 2SD from mean values. Click here for file
