# Supplementary figures and images for: Adaptation of NS cells growth and differentiation to high-throughput screening-compatible plates
Source: BMC Neurosci. 2010 Jan 19;11:7. doi: 10.1186/1471-2202-11-7 (PMC2823757; doi:10.1186/1471-2202-11-7)

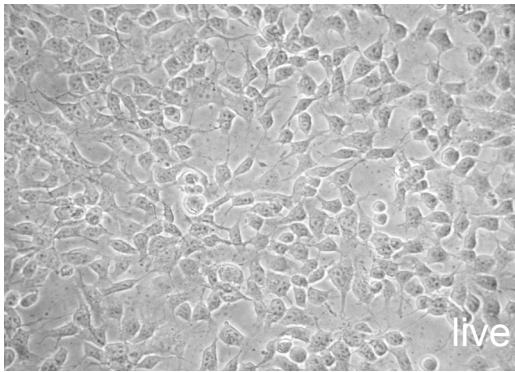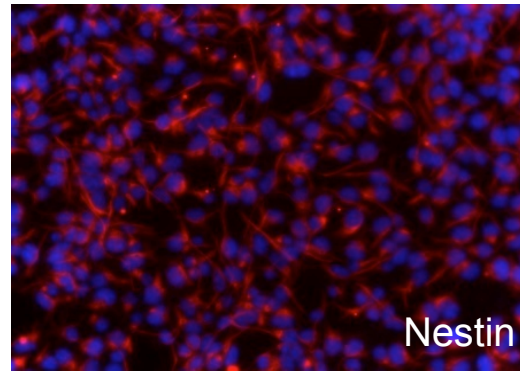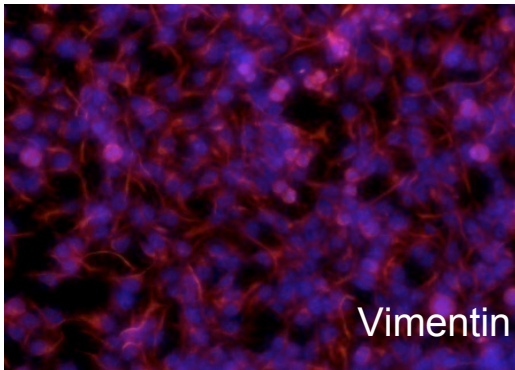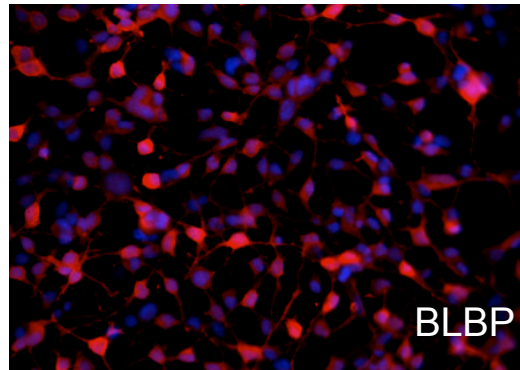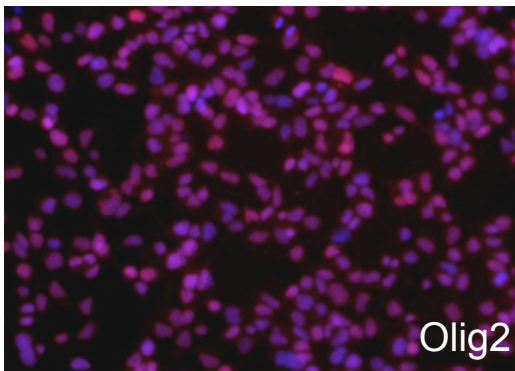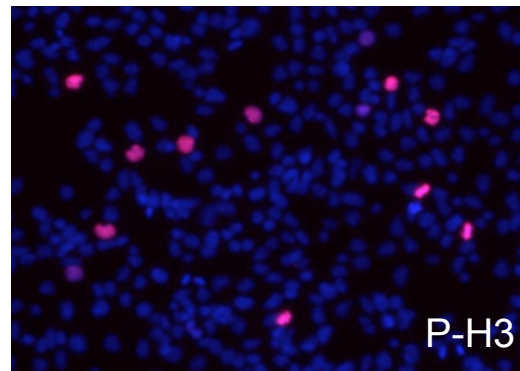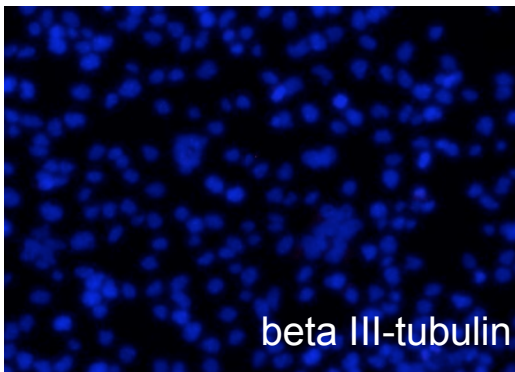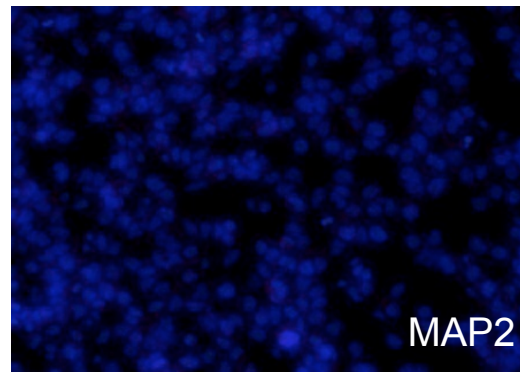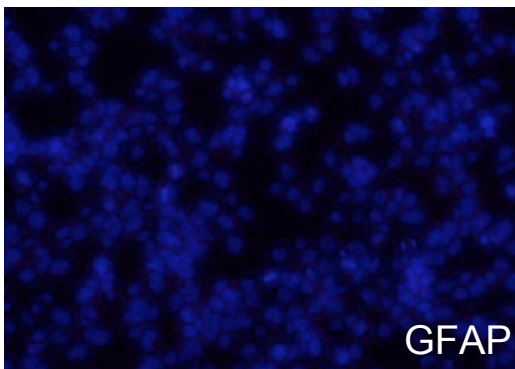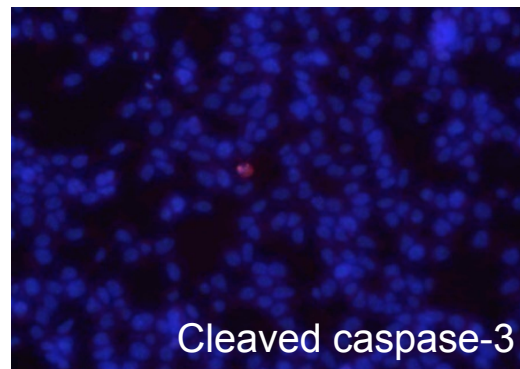

Supplement: Additional file 1 — Adaptation of culture conditions of LC1 cells to microplates. LC1 cells can be cultured in 96-well plates, maintaining the correct expression of NS cells markers (Nestin, Vimentin, BLBP, Olig2, Phospho-Histone3), without differentiation (beta III-tubulin, MAP2 and GFAP absence) or cell death (cleaved caspase-3 absence). [file 1471-2202-11-7-S1.PDF]

**A**

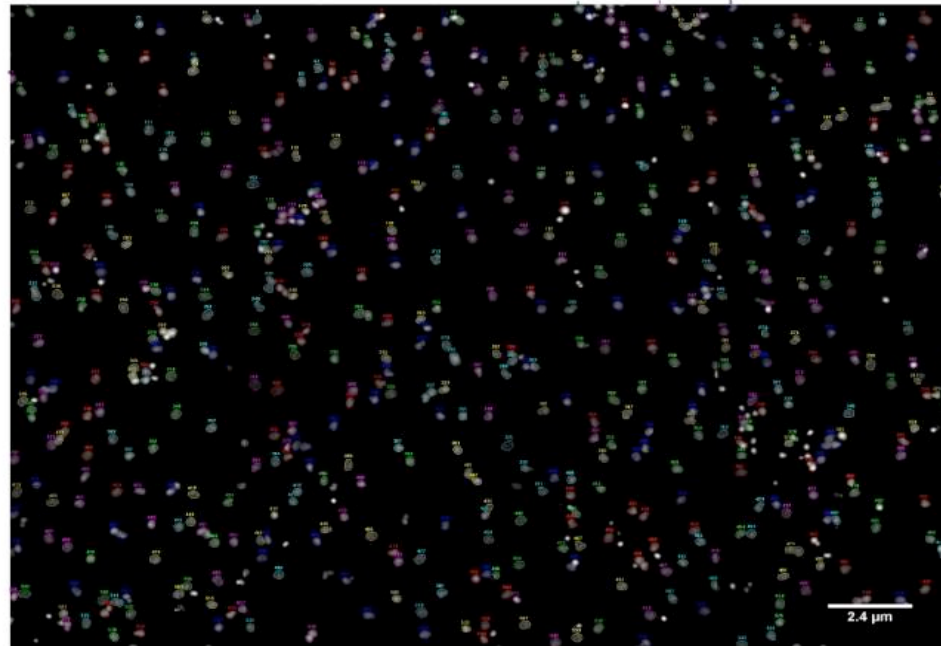

**B**

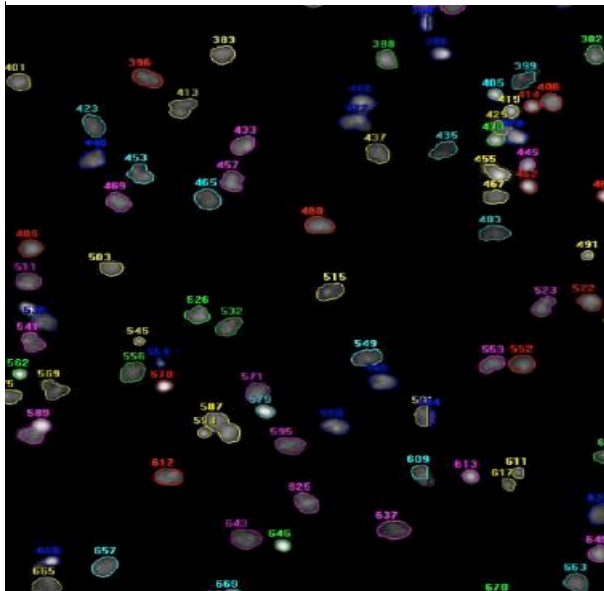

**C**

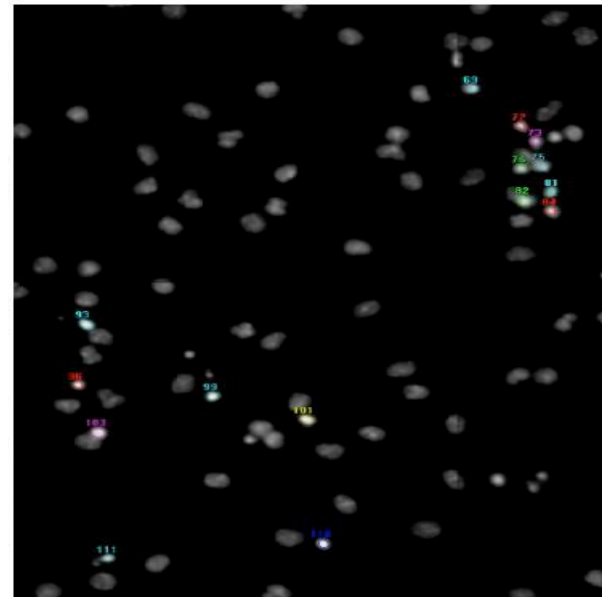

Supplement: Additional file 2 — Examples of the masks designed by the Attovision software. The single colour images, acquired by the BD pathway, were analysed via Attovision software by using specifically designed masks. (A) Example of the mask designed to count DAPI positive alive cells. (B) Mask created to count the total number of cells. (C) Mask able to select picnotic cells only. [file 1471-2202-11-7-S2.PDF]

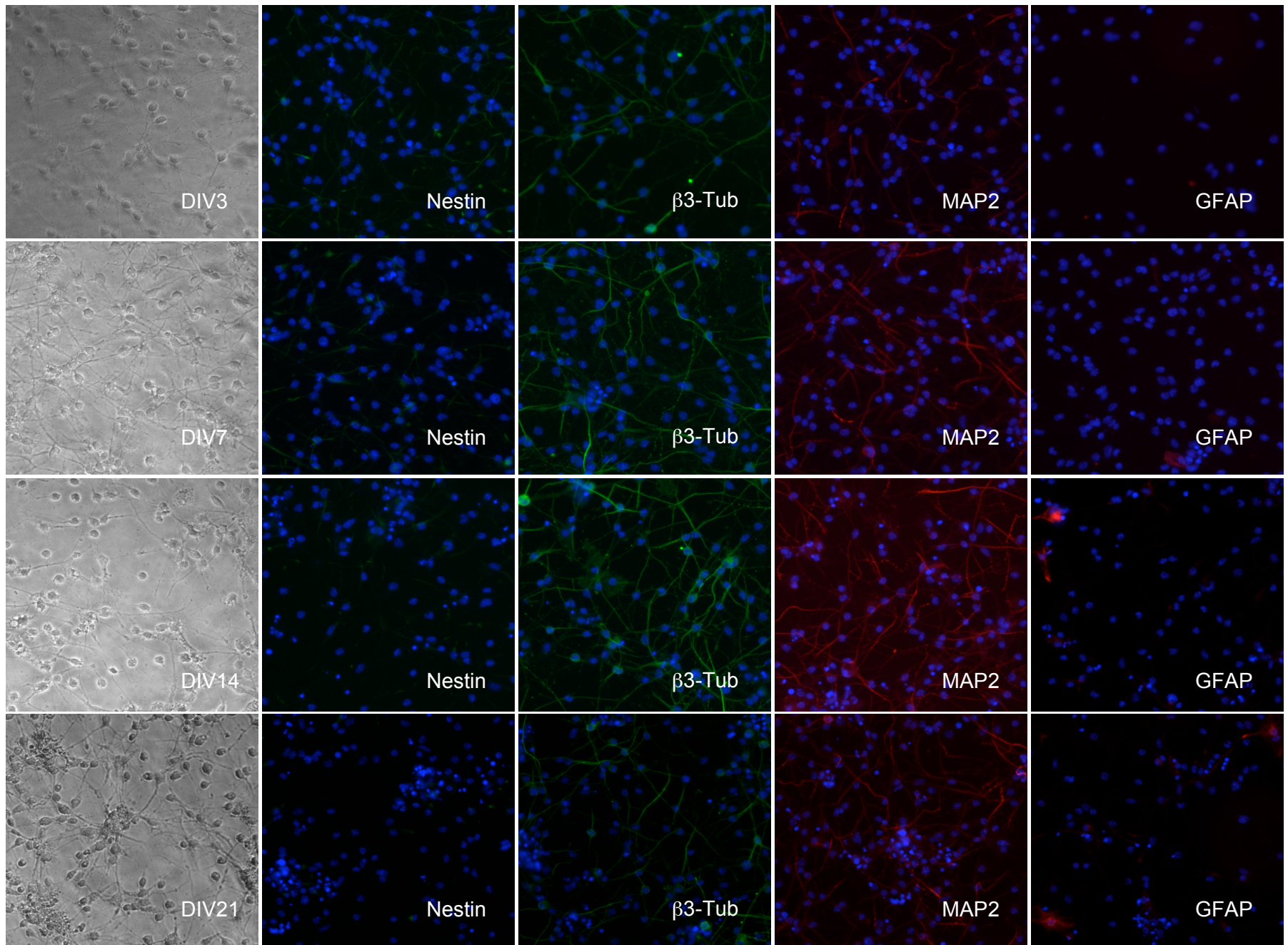

Supplement: Additional file 3 — Differentiating aNS-1 cells progressively acquire neuronal antigenic properties when plated in microplates. Downregulation of NS cells markers such as Nestin (green) and upregulation of neuronal markers beta III-tubulin (green) and MAP2 (red) in aNS-1 cells between DIV3 and DIV21. Low levels of glial marker GFAP (red) were observed at all DIV times. Nuclei were stained with DAPI (blue; all panels). [file 1471-2202-11-7-S3.PDF]

**A**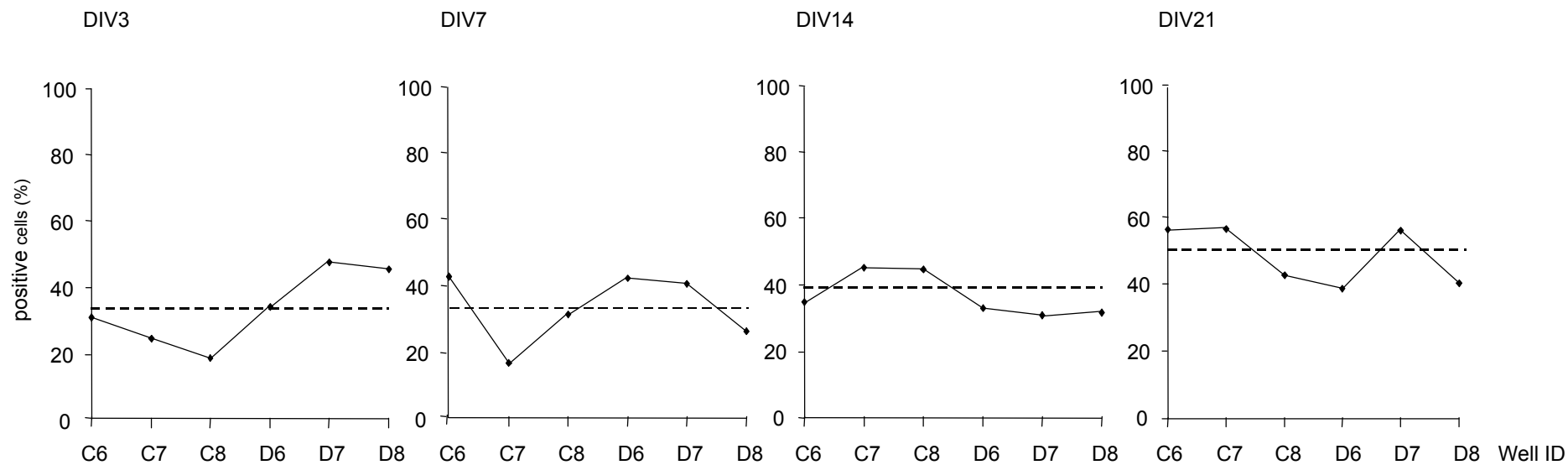**B**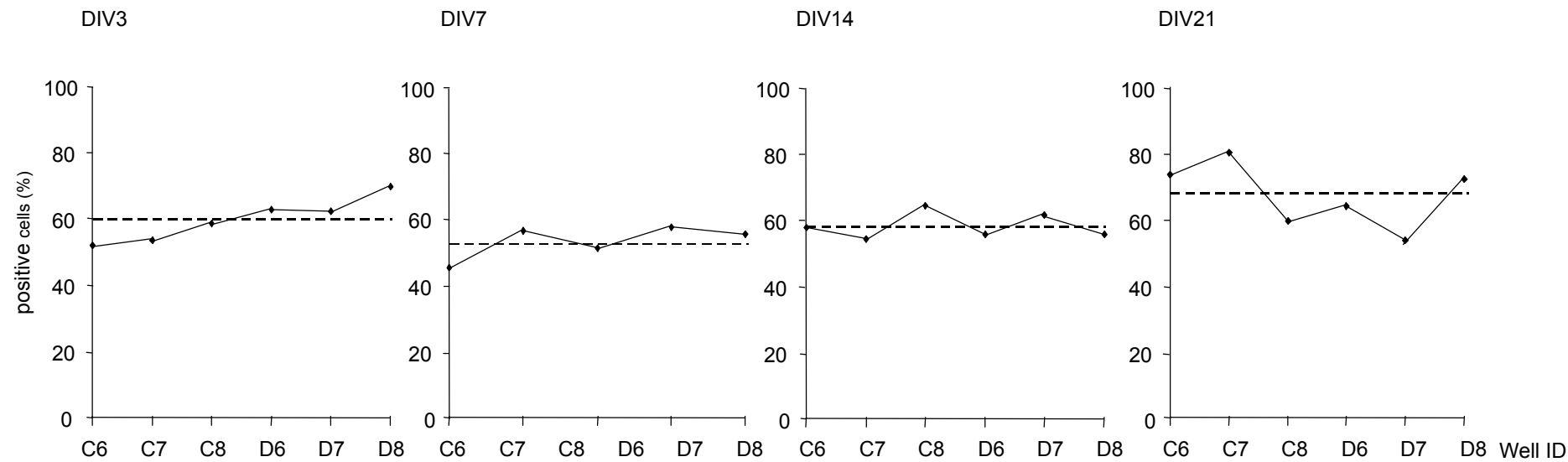

Supplement: Additional file 4 — Intra experiment reproducibility of aNS-1 differentiation procedure. Graphs show relative proportion of alive cells expressing neuronal markers in 6 independent wells from a same 96-well microplate. (A): beta III-tubulin expression. (B): MAP2 expression. Full lines represent mean values. [file 1471-2202-11-7-S4.PDF]

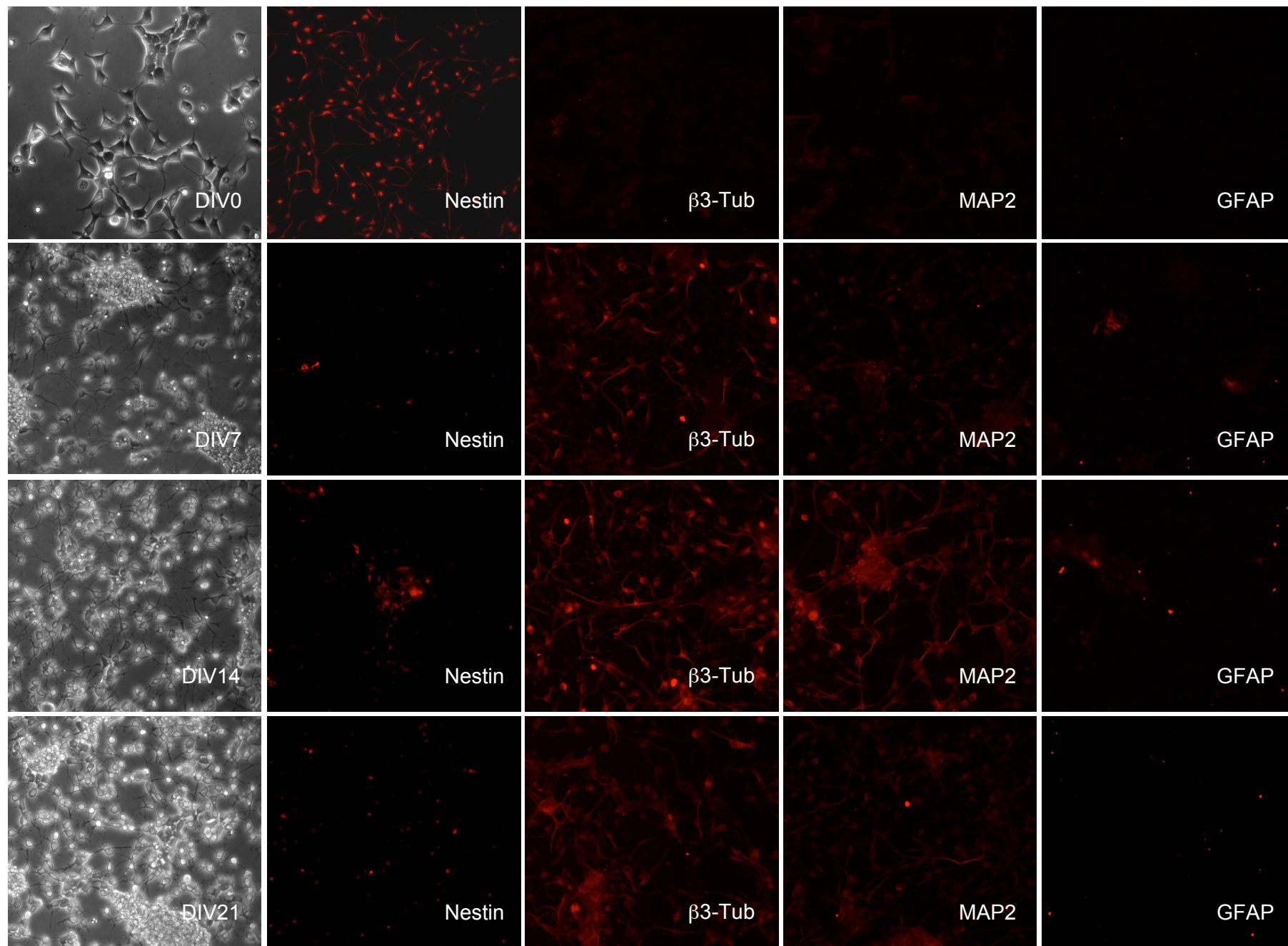

Supplement: Additional file 5 — Differentiation of LC1 cells in 96-well plates. Immunofluorescence experiment showing the expression of markers during the differentiation of LC1 cells in 96-well plates. Most of the differentiating NS cells lose Nestin expression, acquiring a neuronal phenotype (beta III-tubulin and MAP2 immunoreactivity) instead of becoming glial cells (GFAP immunoreactivity). [file 1471-2202-11-7-S5.PDF]

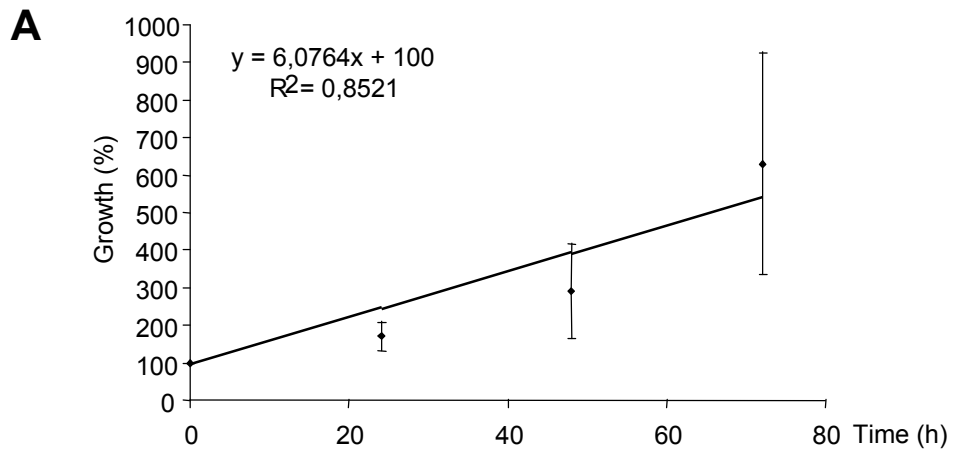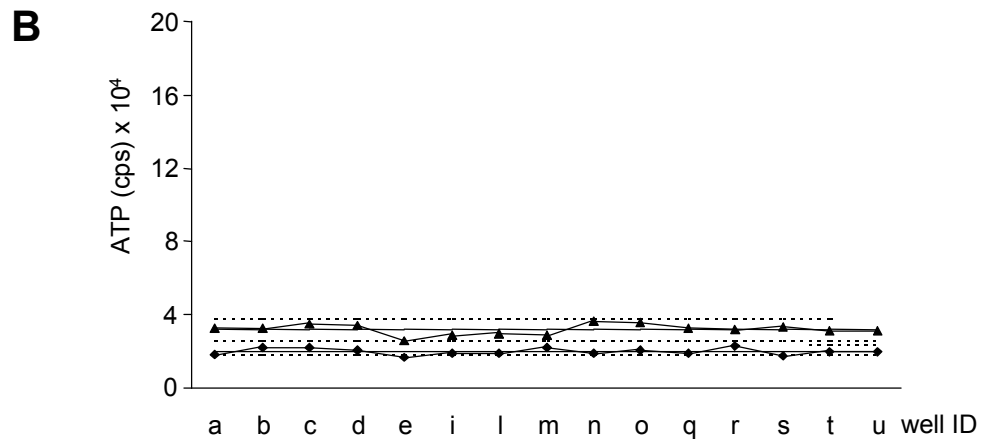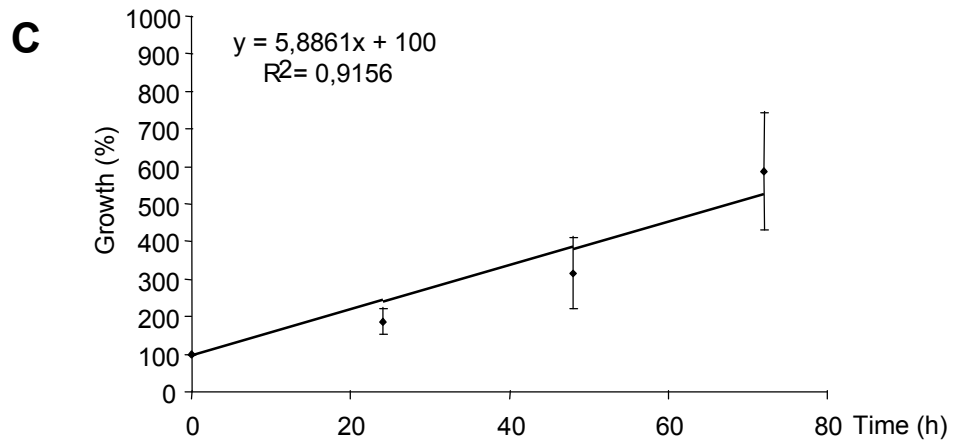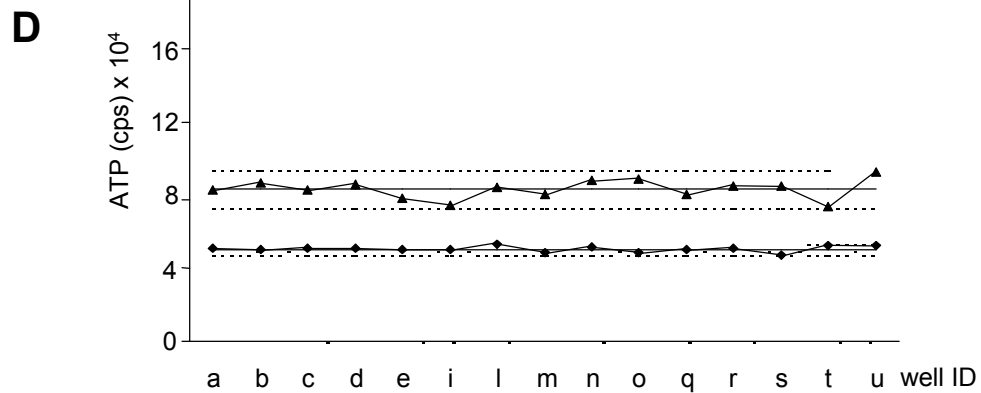

Supplement: Additional file 6 — Cell proliferation assays in 96- and 384-well microplates. (A)and (C)Graphs of representative linear correlations in ATP assay performed in aNS-1 proliferating cells, 4 × 10^3^ cells/well (A) and 16 × 103 cells/well (C) plated in 96-well microplates; (A) and (C): data are expressed as growth (%), referred to the value obtained at t = 0. The data are the mean ± SD from three independent experiments, each one performed in triplicate. (B) and (D): Data dispersion around mean value (full line) from ATP assays performed in 18 wells from 1 × 10^3^ (B) or 2 × 10^3^ (D) cell/well plated in 384-well microplates; upper and lower groups represent 48 h and 24 h data respectively; letters in x axes identify each individual well; dot lines represent 2SD from mean values. [file 1471-2202-11-7-S6.PDF]
